# Prediction of a time-to-event trait using genome wide SNP data

**DOI:** 10.1186/1471-2105-14-58

**Published:** 2013-02-19

**Authors:** Jinseog Kim, Insuk Sohn, Dae-Soon Son, Dong Hwan Kim, Taejin Ahn, Sin-Ho Jung

**Affiliations:** 1 Department of Statistics and Information Science, Dongguk University, Gyeongju 780-714, Korea; 2 Samsung Cancer Research Institute, Samsung Medical Center, Seoul 137-710, Korea; 3 In Vitro Diagnostics Lab., Bio Research Center, Samsung Advanced Institute of Technology, Suwon 449-712, Korea; 4 Department of Medical Oncology and Hematology, Princess Margaret Hospital, University of Toronto, Toronto ON, Canada; 5 Department of Biostatistics and Bioinformatics, Duke University, NC 27710, USA

## Abstract

**Background:**

A popular objective of many high-throughput genome projects is to discover various genomic markers associated with traits and develop statistical models to predict traits of future patients based on marker values.

**Results:**

In this paper, we present a prediction method for time-to-event traits using genome-wide single-nucleotide polymorphisms (SNPs). We also propose a MaxTest associating between a time-to-event trait and a SNP accounting for its possible genetic models. The proposed MaxTest can help screen out nonprognostic SNPs and identify genetic models of prognostic SNPs. The performance of the proposed method is evaluated through simulations.

**Conclusions:**

In conjunction with the MaxTest, the proposed method provides more parsimonious prediction models but includes more prognostic SNPs than some naive prediction methods. The proposed method is demonstrated with real GWAS data.

## Background

A genome-wide association study (GWAS) involves an examination of the entire genome, typically single-nucleotide polymorphisms (SNPs), of different individuals to determine whether any variant is associated with a particular clinical outcome. Many researchers have considered the design and analysis of GWASs with respect to binary clinical outcomes such as case/control or response/non-response ones [1-5].

In clinical cancer research, the primary endpoint of interest is usually a time-to-event trait subject to censoring. In CALGB 80803, for example, germline DNAs are collected, together with time to progression and overall survival data, from 352 advanced pancreatic cancer patients. One objective of an SNP correlative study is to discover SNP markers that are correlated with such time-to-event endpoints.

One of the first objectives of a statistical analysis in a GWAS is the discovery of SNP markers that are associated with a particular trait. The major statistical issue in marker discovery is multiple testing to avoid enlarged type I error probability due to the large number of univariate tests [6,7]. Each prognostic SNP has two or three possible outcomes depending on its genetic model, and the efficiency of a statistical method in associating it with a trait is maximized when the true genetic model is known. For most SNPs, however, the true genetic model is unknown. To identify the true genetic model of each SNP and optimize the association analysis, many researchers have considered some candidate genetic models for a given trait and derived a null distribution of the maximum of test statistics specific to individual genetic models [8,9]. This test is referred to as the MaxTest. These methods have been developed for binary traits such as case/control or response/non-response ones. We develop a MaxTest to identify the genetic model of each SNP when the trait is a survival endpoint, e.g., the time to tumor progression or death.

Another major objective of a GWAS is to predict a trait of interest by using SNPs. Prediction methods using microarray data have been widely investigated [10-12], but cannot be directly applied to SNP-based predictions. The number of SNP markers in genome-wide SNP data far exceeds that of gene markers (or probes) in microarray data, e.g., 1M vs. 20K. In addition, although gene expression data in microarray studies are continuous, genome-wide SNP data are discrete, taking only three different values at most and showing different values depending on the genetic model.

This paper presents a method for predicting a survival outcome that uses genome-wide SNP data but can be easily modified for any type of trait, including binary or continuous outcomes. The proposed method uses the gradient lasso method [13], which has been developed for microarray data. Some investigators fit a prediction model while ignoring the genetic model of each SNP [14]. We also propose a MaxTest associating between a time-to-event trait and a SNP accounting for its possible genetic model and identifies the genetic model of each candidate prognostic SNP by using the proposed MaxTest before fitting a prediction model. The simulation results show that this procedure improves prediction efficiency and prognostic power. For computational efficiency, nonsignificant SNPs are excluded using the MaxTest before starting the gradient lasso. For the facilitation of the proposed MaxTest and prediction method, glcoxphSNP R packages (http://datamining.dongguk.ac.kr/Rlib/glcoxphSNP) are provided.

## Methods

### Genetic Models of SNPs

Suppose that the genotype for an SNP is encoded as AA, AB, or BB. Let *g* denote the number of copies of the B allele. That is, *g*=0, 1 or 2 if the genotype is AA, AB, or BB, respectively. Let *λ*_*g*_(*t*) denote the hazard function of genotype g. Without loss of generality, assume that B is the risk allele in the sense that having B increases the risk of an event. More specifically, assume that *λ*_0_(*t*)≤*λ*_1_(*t*)≤*λ*_2_(*t*) for all *t*≥0. (Note that for some specific diseases, this may not be an appropriate genetic model.) We now consider the following three popular genetic models: 

•*Recessive* model: *λ*_0_(*t*)=*λ*_1_(*t*)<*λ*_2_(*t*).

•*Dominant* model: *λ*_0_(*t*)<*λ*_1_(*t*)=*λ*_2_(*t*).

•*Multiplicative* model: *λ*_2_(*t*)/*λ*_1_(*t*)=*λ*_1_(*t*)/*λ*_0_(*t*), or equivalently *λ*_1_(*t*)=*γλ*_0_(*t*) and *λ*_2_(*t*)=*γ*^2^*λ*_0_(*t*) for *γ*>0.

For a chosen score *c*_*g*_, we consider a proportional hazard model (PHM), *λ*_*g*_(*t*)=*λ*_0_(*t*) exp(*βc*_*g*_). Then Cox’s partial maximum likelihood test has optimal power with (*c*_0_,*c*_1_,*c*_2_)=(0,0,1) for a recessive model, (0,1,1) for a dominant model, and (0,1,2) for a multiplicative model [15]. Note that the PHM is invariant to the linear transformation of the covariate (*c*_0_,*c*_1_,*c*_2_).

### MaxTest

Suppose that we want to test whether an SNP is associated with a given clinical outcome. The test statistic is dependent on the true genetic model of the SNP. At the time of testing, however, we usually have no knowledge of the true genetic model. In this case, a naive approach is to conduct all statistical tests by assuming different genetic models and choose the lowest p-value as the measurement of the association. This approach can lead to an enlarged Type I error because of multiple tests. To adjust for multiple tests, investigators have proposed a method considering the maximum of test statistics with respect to all candidate genetic models under consideration, namely the MaxTest.

Many studies have considered the MaxTest for binary clinical outcomes. Zheng et al. [8] propose a robust ranking method when the underlying genetic model is unknown, namely the MAX-rank test. Conneely and Boehnke [16] propose a method for computing p-values that adjusts for correlated tests and show that the method can improve the accuracy of permutation tests with greater computational efficiency. Li et al. [17] propose a method for approximating the p-value for the MaxTest with or without covariates adjusted for, namely the P-rank test. Li et al. [9] compare the results of the MAX-rank and P-rank tests. Hoggart et al. [18] formulate the problem as variable selection in a logistic regression analysis including a covariate for each SNP and find the posterior mode for shrinkage priors based on a stochastic search on a penalized likelihood function.

We propose a MaxTest for survival endpoints. Here we assign numeric scores to three genotypes based on their genetic model: (*c*_0_,*c*_1_,*c*_2_)=(0,0,1) for a recessive model, (*c*_0_,*c*_1_,*c*_2_)=(0,1,1) for a dominant model, and (*c*_0_,*c*_1_,*c*_2_)=(0,1,2) for a multiplicative model. For a given score *c*_*g*_ assigned to the genotype *g* (=0,1,2) of an SNP, we consider the Cox propotional hazard model,

$$\lambda_{g}(t)=\lambda_{0}(t)\exp(\beta c_{g}).$$

For patient *i* (=1,....,*n*), let *T*_*i*_ and *C*_*i*_ denote the survival and censoring times, respectively. We observe that *X*_*i*_= min(*T*_*i*_,*C*_*i*_) and *δ*_*i*_=*I*(*T*_*i*_≤*C*_*i*_), where *I*(·) indicates the indicator function. In addition, for *t*≥0, let *Y*_*i*_(*t*)=*I*(*X*_*i*_≥*t*) and *N*_*i*_(*t*)=*δ*_*i*_*I*(*X*_*i*_≤*t*) denote the at-risk and event processes, respectively. For a given score, we set *z*_*i*_=*c*_*g*_ if patient *i* has genotype *g*. Let $s_{k}(t)=\overset{n}{\underset{i=1}{\sum }}z_{i}^{k}Y_{i}(t)$, *k*=0,1,2. Then, the partial score test statistic by Cox [19],

$$W=n^{-1/2}\overset{n}{\underset{i=1}{\sum }}\int_{0}^{\infty }\{z_{i}-s_{1}(t)/s_{0}(t)\}dN_{i}(t)$$

is asymptotically normal with mean 0 and variance that can be consistently estimated by

$${\hat{\sigma }}^{2}=n^{-1}\overset{n}{\underset{i=1}{\sum }}\int_{0}^{\infty }\left\{\frac{s_{2}(t)}{s_{0}(t)}-\frac{s_{1}^{2}(t)}{s_{0}^{2}(t)}\right\}dN_{i}(t)$$

under *H*_0_:*λ*_0_(*t*)=*λ*_1_(*t*)=*λ*_2_(*t*) [see, e.g., [20]].

By combining the statistics with respect to the three candidate genetic models, we can derive a MaxTest statistic. Let *W*_*l*_ and ${\hat{\sigma }}_{l}^{2}$ denote the test statistic and the variance estimator with respect to genetic model *l* (=1,2,3). Then we can define the proposed MaxTest as *Q*= max(|*T*_1_|,|*T*_2_|,|*T*_3_|), where $T_{l}=W_{l}/{\hat{\sigma }}_{l}$. Under *H*_0_, $\sigma_{ll^{\prime }}=\text{cov}(W_{l},W_{l^{\prime }})$ is consistently estimated by

$${\hat{\sigma }}_{ll^{\prime }}=n^{-1}\overset{n}{\underset{i=1}{\sum }}\int_{0}^{\infty }\left\{z_{\mathrm{li}}-\frac{s_{l1}(t)}{s_{l0}(t)}\right\}\left\{z_{l^{\prime }i}-\frac{s_{l^{\prime }1}(t)}{s_{l^{\prime }0}(t)}\right\}\frac{Y_{i}(t)}{Y(t)}\mathrm{dN}(t)$$

where *z*_*li*_ and *s*_*lk*_(*t*) denote *z*_*i*_ and *s*_*k*_(*t*), respectively, for genetic model *l*; $Y(t)=\overset{n}{\underset{i=1}{\sum }}Y_{i}(t)$, $N(t)=\overset{n}{\underset{i=1}{\sum }}N_{i}(t)$. Let $\hat{\Sigma }={\left({\hat{\rho }}_{ll^{\prime }}\right)}_{1\leq l,l^{\prime }\leq 3}$, where ${\hat{\rho }}_{ll^{\prime }}={\hat{\sigma }}_{ll^{\prime }}/{\hat{\sigma }}_{l}{\hat{\sigma }}_{l^{\prime }}$. Then we can obtain the critical value of *Q* by a numerical method or a simulation method from the $N(0,\hat{\Sigma })$ distribution. This is a survival trait counterpart for the MaxTest with a binary trait, as discussed in several studies [9,21].

We can construct an alternative test based on the quadratic form $W^{2}=S^{T}{\hat{\Sigma }}^{-1}S$, where *S*=(*T*_1_,*T*_2_,*T*_3_)^*T*^. In addition to recessive, dominant, and multiplicative genetic models, we can consider other models to develop a test statistic to measure the relationship between an SNP and a survival trait. For example, we may consider the long-rank test based on the one-way ANOVA in [22] or the test based on the Wilcoxon Rank-Sum test in [23], which require no specific genetic model assumptions. In particular, the ANOVA-type test is a reasonable option if the monotone trend in genotypes g = 0, 1, and 2 is doubtful.

### Cox model with a lasso penalty

In an analysis using SNP data, we may face a problem in which the number of SNPs exceeds the size of data, that is, *m*≫*p*, which frequently occurs even when a smaller number of SNPs are selected through a prescreening step. This may lead to a serious collinearity problem when directly applying the partial likelihood estimation to the Cox model. To address this problem, Tibshirani[24] estimates the parameters of the Cox model under the *L*_1_ constraint as follows:

$$\hat{\beta }=\arg\underset{\beta ,s}{\max}l(\beta ),\text{subject to}\overset{m}{\underset{j=1}{\sum }}|\beta_{j}|\leq s,$$

where *l*(·) is the partial likelihood function [19].

The above optimization problem is suitable for reducing the dimension of covariates but is computationally difficult because the *L*_1_ objective function is not differentiable. To address this computational problem, previous studies have proposed many algorithms [13,24-26]. Tibshirani[24] proposes an algorithm using quadratic programming within an iterative procedure. Gui and Li[25] propose an LAS-Cox procedure applying the Choleski decomposition and the LARS procedure. However, these algorithms can be computationally burdensome and sometimes fail to converge to an optimum because they involve quadratic programming and/or matrix inversions. Sohn et al. [13] propose glcoxph for the Cox model by using the gradient lasso algorithm in [27]. This glcoxph employs a coordinate-wise gradient decent with a deletion step and requires only univariate optimization in each iteration. Its convergence speed is almost independent of the number of input variables, and it does not require a matrix inversion, which makes it scalable to high-dimensional data and allows it to converge to a global optimum faster. glmpath [26] provides the entire penalization path for the Cox model in a forward stagewise manner. Because it requires matrix inversions only for active sets, it is faster and more stable than other methods. Sohn et al. [13] provided a comparative analysis using real and simulated data and show that the gradient lasso algorithm outperforms glmpath in analyzing high-dimensional survival data in terms of the sparsity, predictability, and computational efficiency of the final prediction model. Therefore, the following gradient lasso algorithm can be a useful alternative for fitting the Cox model to predict the survival time of patients based on high-dimensional SNP data: 

1. **Initialize**: ***β***=**0** and *k*=0.

2. **Do** until convergence 

(a) **Addition:**

(i) Compute the gradient ∇*l*(***β***).

(ii) Find the $\overset{j}{\gamma }$ maximizing |*∂l*(***β***)/*∂β*_*j*_| for *j*=1,…,*p* and $\hat{\gamma }=-s\times \mathrm{sign}(\mathrm{\partial l}(\beta )/\partial \beta_{ĵ})$.

(iii) Let ***v*** be a *p*-dimensional vector such that its $\overset{j}{\gamma }$-th element $\hat{\gamma }$ and other elements are zeros.

(iv) Find $\hat{\alpha }=\arg\underset{\alpha \in [0,1]}{\min}l\left((1-\alpha )\beta +\alpha v\right).$

(v) Update $\beta =(1-\hat{\alpha })\beta +\hat{\alpha }v.$

(b) **Deletion:**

(i) Calculate ***h***_*σ*_=−∇*l*(***β***_*σ*_)+*θ*_*σ*_∇*l*(***β***_*σ*_)^*T*^*θ*_*σ*_/|*σ*|, where *σ*={*j*:*β*_*j*_≠0}.

(ii) Find $\hat{\delta }=\arg\underset{\delta \in [0,U]}{\min}l(\beta +\delta h),$ where $h=\left(\begin{array}{c} h_{\sigma }\\ 0 \end{array}\right)$ and *U*= min*k*∈*σ*{−*β*_*k*_/*h*_*k*_:*β*_*k*_*h*_*k*_<0}.

(iii) Update $\beta =\beta +\hat{\delta }h$.

(c) Set *m*=*m*+1.

3. **Return*β***.

### Proposed algorithm for predicting a survival trait

We propose a new algorithm for fitting a Cox regression model using SNP data. The proposed algorithm consists of the following four steps: We (i) select significant SNPs by the MaxTest, as in Section “Example using real data”, (ii) convert these SNPs into corresponding scores by genetic models identified by the MaxTest, (iii) standardize these scores, and (iv) apply the gradient lasso algorithm [13] to selected SNPs. We summarize the algorithm in greater detail as follows: 

1. Read in the clinical data {(*X*_*i*_,*δ*_*i*_),*i*=1,...,*n*} and SNP data {(*s*_*i*1_,...,*s*_*im*_),*i*=1,...,*n*}, where *s*_*ij*_ denotes the number of *B* alleles for SNP *j* (=1,...,*m*).

2. For SNP *j* (=1,...,*m*), calculate the variance and covarince matrix ${\hat{\Sigma }}_{j}$, and generate the null distribution of the MaxTest as follows. 

(a) For *b*=1,...,*B* (=100,000, say), generate $\left(t_{1j}^{\left(b\right)},t_{2j}^{\left(b\right)},t_{3j}^{\left(b\right)}\right)$ from $N(0,{\hat{\Sigma }}_{j})$.

(b) Let $q_{j}^{\left(b\right)}=\max(|\overset{\left(b\right)}{\underset{1j}{t}}|,|t_{2j}^{\left(b\right)}|,|t_{3j}^{\left(b\right)}|)$ for *b*=1,...,*B*.

3. For SNP *j* (=1,...,*m*), 

(a) Using original data, calculate the test statistics (*T*_1*j*_,*T*_2*j*_,*T*_3*j*_), the MaxTest statistic *q*_*j*_= max(|*T*_1*j*_|,|*T*_2*j*_|,|*T*_3*j*_|), and two-sided p-values *p*_1*j*_,*p*_2*j*_,*p*_3*j*_ from the marginal test for respective genetic models.

(b) Approximate the p-value of the MaxTest by

$$p_{j}=B^{-1}\overset{B}{\underset{b=1}{\sum }}I(q_{j}^{\left(b\right)}\geq q_{j})$$

4. SNP screening: Select *J* (<<*m*) SNPs with *p*_*j*_<*α* for specified a *α* (=0.01, say).

5. For selected SNPs *j* (=1,...,*J*), identify the genetic model (1, 2, 3) by the lowest marginal p-value from *p*_1*j*_,*p*_2*j*_,*p*_3*j*_ or the largest test statistic from *T*_1*j*_,*T*_2*j*_,*T*_3*j*_.

6. For patient *i* (=1,...,*n*), define covariates (*z*_*i*1_,...,*z*_*iJ*_) by the identified genetic model and the corresponding score.

7. Standardize the covariates:

$$z_{\mathrm{ij}}^{\prime }=\frac{z_{\mathrm{ij}}-{\bar{z}}_{j}}{s_{j}},$$

where ${\bar{z}}_{j}=n^{-1}\overset{n}{\underset{i=1}{\sum }}z_{\mathrm{ij}}$ and $s_{j}^{2}=n^{-1}\overset{n}{\underset{i=1}{\sum }}{\left(z_{\mathrm{ij}}-{\bar{z}}_{j}\right)}^{2}$.

8. Apply the gradient lasso to the Cox regression model with response data {(*X*_*i*_,*δ*_*i*_),*i*=1,...,*n*) and standardized covariates $\left\{(z_{i1}^{\prime },\mathrm{...},z_{\mathrm{iJ}}^{\prime }),i=1,\mathrm{...},n\right\}$.

## Results and discussion

### Simulation study

We provide a simulation study. The data generation scheme is as follows: We generate SNP data *z*_1_,...,*z*_*m*_ from *N*(0,1) random variables with an AR(1) correlation structure with the autocorrelation coefficient *ρ*(≥0), *x*_1_,...,*x*_*m*_. Due to linkage disequilibrium, SNPs which lay in close vicinity within chromosomes tend to have a stronger association. In this sense, an AR(1) correlation structure is a reasonable correlation structure for the continuous random variables generating SNP data. Let *x*_1_=*ε*_1_ and $x_{j}=\rho x_{j-1}+\sqrt{1-\rho^{2}}\varepsilon_{j}$ for *j*=2,...,*m*, where *ε*_1_,...,*ε*_*m*_∼IID*N*(0,1) random numbers. The cutoff values for *x*_*j*_ for generating *z*_*j*_ determine allele frequency. For each SNP, let *f*_1_,*f*_2_,*f*_3_ (*f*_1_+*f*_2_+*f*_3_=1) denote the frequency of AA, AB, and BB genotypes, respectively, where B denotes the risk allele. Note that marginally *x*_*j*_∼*N*(0,1). The true model for the survival times is given as

$$\Lambda (t)=\Lambda_{0}(t)\exp\left(\overset{D}{\underset{j=1}{\sum }}\beta_{j}z_{\mathrm{ij}}\right),$$

where D denotes the number of prognostic SNPs.

For the experiment, we set *m*=1000, *n*=200, *D*=6, *ρ*=0 or 0.3, *β*_*j*_=0.8 (*j*=1,...,*D*), and a uniform censoring distribution for 15% or 30% of censoring. All six prognostic SNPs have (*f*_1_,*f*_2_,*f*_3_)=(.25,.5,.25). SNP 1 and SNP 4 have a dominant model; SNP 2 and SNP 5, a recessive model; and SNP 3 and SNP 6, a multiplicative model. Each of the remaining 994 SNPs has (AA,AB,BB) with (*f*_1_,*f*_2_,*f*_3_)=(1/3,1/3,1/3).

To evaluate the performance of the proposed method, we generate 200 random samples and divide them into a training set (100 samples) and a test set (100 samples). We calculate the MaxTest p-value of each SNP by using B=100,000 permutations from the training set and identify the genetic model for each SNP. We select SNPs with p-values less than *α*=0.01 and convert selected SNPs into corresponding scores by their genetic models. We apply the gradient lasso to the selected SNPs to fit the prediction model. Let SNPs *j* (=1,...,*K*) be included in the fitted prediction model with corresponding regression estimates ${\hat{\beta }}_{1},\mathrm{...},{\hat{\beta }}_{K}$. Then we can define the risk score for sample *i* as $r_{i}={\hat{\beta }}_{1}z_{i1}+\cdots +{\hat{\beta }}_{K}z_{\mathrm{iK}}$. Using the median risk score from the test set as a cutoff value, we divide the patients in the test set into high- and low-risk groups. We apply a two-sample log-rank test to compare the survival distribution between these two risk groups. We repeat this procedure 100 times and count the number of SNPs and that of prognostic SNPs included in each fitted prediction model by the gradient lasso. We summarize the distribution of log-rank p-values from the test set, and for comparison purposes, we consider the prediction methods by assuming that all *m* SNPs have the same genetic model.

Table 1 reports the mean number of SNPs and that of prognostic SNPs included in the fitted prediction model, recovery rate, and the means (and standard deviations) of the log-rank p-value from the test set for the proposed method and the methods assuming a recessive, dominant, or multiplicative model for all SNPs under various simulation settings. We define recovery rate as the ratio of mean number of selected prognostic SNP to the mean number of selected SNP as in Sohn et al. [13]. The proposed method tends to result in prediction models with a smaller number of SNPs but a larger number of prognostic SNPs than the approaches assuming a specific genetic model for all SNPs (i.e., recessive, dominant, and multiplicative methods in the table). The recovery rates of the proposed method are higher than those of the methods based on a pre-specified model (recessive, dominant, and multiplicative). Among the three methods assuming a specific genetic model for all SNPs, the one assuming a multiplicative model shows the best performance in terms of the number of prognostic SNPs included in the final prediction model. In addition, the proposed method outperforms the recessive, dominant, and multiplicative methods in terms of the log-rank p-value and results in fitted prediction models with a smaller number of SNPs but a larger number of prognostic SNPs with 15% compared to 30% censoring. According to sample size (*n*) and the effect size (*β*), the mean number of SNPs and that of prognostic SNPs selected by the proposed method at *ρ*=0 and 30% censoring is shown in Table 2. The mean number of prognostic SNPs a little bit increases as *β* increase and the mean number of prognostic SNPs increases as *n* increases. The recovery rate increases as *β* or *n* increases.

**Table 1:** Mean numbers of SNPs and prognostic SNPs included in fitted prediction models, recovery rate and means/standard deviations of the log-rank p-value from test sets for the proposed method and methods assuming recessive, dominant, or multiplicative models for all SNPs

**Censoring**	***ρ***	**Genetic**	**Mean number**	**Mean number of**	**Recovery**	**Mean (SD) p-value**
		**model**	**of selected**	**of selected**	**rate**	**of the log-rank**
			**SNPs**	**prognostic SNPs**		**test**
30%	0	Proposed	6.72	5.05	0.75	<0.0001 (<0.0001)
		Recessive	8.03	4.01	0.50	0.0052 (0.0018)
		Dominant	6.66	3.85	0.58	<0.0001 (<0.0001)
		Multiplicative	7.72	4.95	0.64	0.0004 (0.0003)
	0.3	Proposed	6.51	4.83	0.74	0.0001 (0.0001)
		Recessive	7.73	3.83	0.50	0.0045 (0.0016)
		Dominant	6.58	3.66	0.56	0.0011 (0.0007)
		Multiplicative	7.52	4.72	0.63	0.0006 (0.0004)
15%	0	Proposed	6.65	5.18	0.78	<0.0001 (<0.0001)
		Recessive	8.59	4.19	0.49	0.0028 (0.0011)
		Dominant	6.69	3.88	0.58	<0.0001 (<0.0001)
		Multiplicative	7.96	4.98	0.63	0.0005 (0.0005)
	0.3	Proposed	6.37	4.98	0.78	<0.0001 (<0.0001)
		Recessive	7.88	3.94	0.50	0.0048 (0.0028)
		Dominant	6.38	3.74	0.59	0.0011 (0.0011)
		Multiplicative	7.55	4.89	0.65	0.0001 (<0.0001)

**Table 2:** **Mean number of SNPs and prognostic SNPs included in the fitted prediction models, recovery rate and means/standard deviations of the log-rank p-values from the test set for the proposed method at *ρ* = 0 and censoring = 30%**

**n**	***β***	**Mean number of**	**Mean number of**	**Recovery rate**	**Mean (SD) p-value**
		**selected SNP**	**selected prognostic SNP**		**of the log-rank test**
200	0.8	6.72	5.05	0.75	<0.0001 (<0.0001)
	1	6.13	5.18	0.85	<0.0001 (<0.0001)
	2	5.60	5.17	0.92	<0.0001 (<0.0001)
300	0.8	6.18	5.53	0.89	<0.0001 (<0.0001)
400	0.8	5.89	5.72	0.97	<0.0001 (<0.0001)

### Example using real data

We apply the proposed method to the GWAS data in Choi et al. [28], who provide a GWAS of 119 patients with normal karyotype acute myeloid leukemia (AML-NK) by using Affymetric Genome-Wide Human SNP Arrays 6.0 (San Diego, CA, USA). We exclude those SNPs with missing genotype data for any patient. We also exclude those SNPs with only one genotype for the 119 patients. The final data set for the analysis includes *m* = 251, 748 autosomal SNPs from *n* = 119 patients. The primary endpoint in this analysis is event-free survival (EFS), which is defined as the interval between the registration and the end of induction chemotherapy for patients showing no complete response (CR), a relapse after achieving a CR to induction chemotherapy, or death.

**Figure 1:**
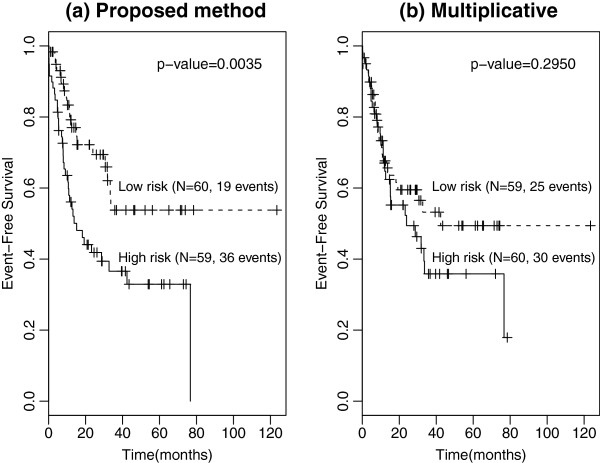
Kaplan-Meier curves for high- and low-risk groups classified by the LOOCV procedure.

We employ the leave-one-out cross-validation (LOOCV) procedure to evaluate the predictive performance of the proposed method for the data set. From a training set of size *n*−1 = 118, we calculate the MaxTest p-value of each SNP based on *B*=100,000 permutations, select *J* candidate SNPs with p-values less than *α*=0.01 by MaxTest, and apply the gradient lasso to candidate SNPs to obtain a prediction model. Using the median risk score for the patients in the training set, we allocate those patients who are left out for the validation to the high- or low-risk group. We repeat this procedure *n* times and calculate the log-rank p-value to compare the EFS between the two risk groups. Figure 1(a) shows the Kaplan-Meier curves for the high- and low-risk groups classified by the LOOCV procedure. The five-year EFS rate for the low-risk group (n=60, 53.8%) is much higher than that for the high-risk group (n=59, 32.9%) with the estimated hazard ratio of 0.446 (95 % CI, 0.256-0.778), and the log-rank p-value is 0.0035.

A standard approach may be to fit a prediction model assuming a multiplicative genetic models for all SNPs, e.g. Tan et al. [29]. We analyzed this data set using the same method as above except that all SNPs were assumed to have a multiplicative model. Figure 1(b) displays the LOOCV results. Note that the fitted prediction models do not significantly partition the test set into high- and low-risk groups by ignoring the possible genetic models.

We also apply our prediction procedure to the whole data set with *n*=119. Using *α*=0.01, we select *J*=1122 candidate SNPs, among which 444 (39.6%) are shown to have a recessive model, 463 (41.3%) a dominant model, and 215 (19.2%) a multiplicative model. By applying the gradient lasso to the selected 1122 SNPs, we obtain the final prediction model including *k*=24 SNPs. Table 3 lists the RS IDs, the chromosome numbers, the base-pair position and the gene name of the 24 SNPs included in the final prediction model. For each of the 24 SNPs, we report the genetic model (=1 for recessive model, =2 for dominant model, and =3 for multiplicative model) identified by the MaxTest, the marginal MaxTest p-value and number of times (frequency) that each SNP is included in the prediction models during the LOOCV. Note that the first four SNPs in Table 2 are included in all 119 prediction models during LOOCV.

**Table 3:** List of 24 SNPs selected by the proposed method from the whole data set of 119 samples, their MaxTest p-values, genetic models, the number of times selected by prediction models fitted during the LOOCV procedure

**RS ID**	**Chr**	**Position**	**Gene name**	**Genetic**	**P-value**	**Frequency**
				**model *g***		
rs1030254	16	60696651	LOC644649, CDH8, LOC729159	3	0.00009	119
rs1030252	16	60696869	LOC644649, CDH8, LOC729159	2	0.00010	119
rs10798122	1	187584699	PLA2G4A, FAM5C	1	0.00048	119
rs10026207	4	186039201	HELT, SLC25A4	3	0.00233	119
rs13333329	16	1695776	CRAMP1L	3	0.00015	117
rs2132183	3	84966867	LOC440970,CADM2	3	0.00149	117
rs1950400	14	27105035	MIR4307,NOVA1	2	0.00040	115
rs2155777	11	133290007	OPCML	3	0.00142	113
rs1677914	12	78274425	NAV3	2	0.00283	106
rs1476847	18	9834599	RAB31	1	0.00029	102
rs7614596	3	84986027	LOC440970,CADM2	2	0.00020	100
rs2648117	4	186787096	SORBS2	3	0.00856	90
rs1851317	15	35077786	GJD2,ACTC1	1	0.00999	88
rs3790217	20	19441650	SLC24A3	2	0.00728	85
rs4902990	14	72618432	RGS6	2	0.00004	81
rs9482583	6	125318379	RNF217	3	0.00847	79
rs3020444	14	64791013	ESR2	3	0.00288	77
rs10851869	15	74331083	PML	2	0.00036	65
rs11986200	8	22698209	PEBP4	1	0.00222	63
rs11260756	1	16759616	SPATA21	1	0.00827	63
rs4968415	17	60264240	MED13,TBC1D3P2	1	0.00075	62
rs12416722	11	133300460	OPCML	1	0.00067	59
rs626266	12	72888187	TRHDE	2	0.00070	52
rs16852300	2	167414424	SCN7A,XIRP2	3	0.00513	33

The RGS6 gene (rs4902990) is associated with treatment outcomes in AML-NK patients. RGS6, a regulator of G-protein signaling 6, modulates the G-protein function in the signaling pathway by activating the intrinsic GTPase activity of alpha subunits [30,31]. An SNP on RGS6 has been found to modulate the risk of bladder cancer [32]. In addition, it is known that RGS6 induces apoptosis through a mitochondrial-dependent pathway, which implies that RGS6 may be involved in cancer progression [29]. Further, membrane drug transporters, including SLC25A4 (rs10026207) and SLC24A3 (rs3790217), are known to be associated with event-free survival. SLC25A4, solute carrier family 25 (mitochondrial carrier; adenine nucleotide translocator; ANT1), member 4, is known to interact with the Bcl-2-associated X protein, which is involved in the apoptosis pathway [33,34]. The rs10798122 SNP on family with sequence similarity 5, member C, FAM5C, is selected by the proposed model. A loss of hypermethylated FAM5c is known to be associated with the development of tongue squamous cell carcinoma or gastric cancer [35,36].

## Conclusions

We have proposed a prediction method for a survival endpoint using SNPs. The paper also proposes a MaxTest to screen out nonprognostic SNPs and identify genetic model of prognostic SNPs. The simulation results indicate substantial prognostic power for the proposed prediction method. Noteworthy is that, in conjunction with the MaxTest, the proposed method provides more parsimonious prediction models with more prognostic SNPs than those prediction methods ignoring the true genetic model of prognostic SNPs. We apply real GWAS data to patients with acute myeloid leukemia and find that the proposed method provides a prediction model that can efficiently classify the patients into high- and low-risk groups by using a small number of SNPs that are known to be biologically informative. Although the proposed method is limited to the prediction of time-to-event traits, it can be easily extended to a wide range of traits, including dichotomous or continuous ones.

## Competing interests

The authors declare no conflict of interest

## Author contributions

JK and IS performed the statistical analysis and wrote the manuscript. DS and TA supported the research. DHK provided a biological interpretation of the statistical analysis. SJ proposed the research project. All authors read and approved the final manuscript.
